# Reductions in anxiety and depression symptoms in a subset of outpatients with problematic substance use who received ketamine-assisted psychotherapy: a two-year retrospective chart review

**DOI:** 10.3389/fpsyt.2023.1160442

**Published:** 2023-08-30

**Authors:** Emily Whinkin, Therry Rose J. Eparwa, Michelle C. Julseth, Andrea Schneider, Sunil K. Aggarwal

**Affiliations:** ^1^Advanced Integrative Medical Sciences Institute, Seattle, WA, United States; ^2^College of Nursing, Seattle University, Seattle, WA, United States

**Keywords:** ketamine, ketamine-assisted psychotherapy, substance use disorders, anxiety, depression, psychometrics, spiritual distress

## Abstract

**Objective:**

Assess changes in symptoms of anxiety, depression, and psychosocial or spiritual distress before and after ketamine-assisted psychotherapy (KAP) in individuals with problematic substance use (PSU).

**Methods:**

A retrospective chart review was performed on participant data from two five-year prospective outcomes studies: the AIMS Medical Outcomes Study (AMOS) and the AIMS Cancer Outcomes Study (ACOS). The efficacy of KAP for anxiety, depression, and psychosocial or spiritual well being was assessed in patients with current, past, or high risk of substance use disorder. Validated psychometrics utilized were Generalized Anxiety Disorder-7 (GAD-7), Patient Health Questionnaire-9 (PHQ-9), and the National Institute of Health - Healing Experiences of All Life Stressors (NIH-HEALS) questionnaires.

**Results:**

Between November 1, 2020 and October 31, 2022, a total of 18 patients identified with problematic substance use completed at least one KAP session and at least one baseline and post-KAP metric questionnaire. The PSU subpopulation average score changes were as follows: GAD-7 (-6.71 ± 9.15, *n* = 14); PHQ-9 (-7.44 ± 5.42, *n* = 16); and NIH-HEALS (5.13 ± 13.64, *n* = 15). The average score changes for the KAP population of enrolled subjects were as follows: GAD-7 (-2.45 ± 6.01, *n* = 104); PHQ-9 (-3.02 ± 6.01, *n* = 111); and NIH-HEALS (2.93 ± 11.91, *n* = 86). A comparison of average score changes (*p* < 0.05) between the PSU subpopulation and KAP population were as follows: GAD-7 (0.0219, 95% C.I. 1.37-8.11); PHQ-9 (0.0062, 95% C.I. 1.28-7.56); and NIH-HEALS (0.5197, 95% C.I. 8.96-4.56). For patients with PSU, results demonstrate statistically significant improvements in anxiety and depression symptoms after at least one KAP session. Average NIH-HEALS scores increased, though not by a statistically significant amount. Compared to the general population of enrolled KAP patients during this period, patients with PSU reported significantly greater average reductions in GAD-7 and PHQ-9 scores.

**Conclusion:**

Undergoing one to six ketamine-assisted psychotherapy (KAP) sessions was associated with improved anxiety and depression ratings in patients with problematic substance use. Two-thirds of participants also experienced improved psychosocial and spiritual well-being. The use of KAP may be important to consider as a therapy for reducing anxiety and depression symptoms in patients with problematic substance use.

## 1. Introduction

### 1.1. Problematic substance use

The British Columbia Ministry of Health described a comprehensive paradigm of assessment and response to addictions and substance use in 2004, introducing the terminology of ‘problematic substance use.’ Problematic substance use (PSU) includes individuals who have potentially harmful substance use behaviors or patterns that are not clinical disorders (e.g., driving while impaired, using substances while pregnant) *and* individuals who have substance use disorders as defined by the *Diagnostic and Statistical Manual of Mental Disorders* (5th ed.; DSM-5) (1). This particular framework acknowledges that there is a spectrum to substance use instances or patterns, from ‘beneficial use’ to ‘non-problematic use’ to ‘problematic use.’ Substance use disorders are considered the extreme and most damaging end of the spectrum. For the purposes of this study, those with PSU include individuals with clinician-identified substance use disorder and individuals with self-identified or clinician-identified potentially harmful substance use. For the purposes of this manuscript we use ‘high risk’ and ‘problematic’ interchangeably in regard to the spectrum of substance use in this urban outpatient population.

### 1.2. Prevalence of substance use disorders and other mental illnesses

Substance use disorder (SUD) is a prevalent and difficult-to-treat condition that is made more complicated by comorbid mood disorders and biopsychosocial barriers to healing. From 2020 to 2021, an estimated 46.3 million people (16.5 percent of the population) aged 12 or older were living with a diagnosed SUD in the United States. Of this group, 43.7 million (15.6 percent) needed SUD treatment but only 4.1 million people (1.5 percent) received treatment specifically for SUD (2).

In 2021, there were an estimated 26 million persons (9.3 percent of the population) aged 12 or older who had a major depressive episode (MDE) in the past year, for which 14.6 million (5.2 percent) received treatment. Among adults aged 18 or older, 57.8 million persons (22.8 percent) had one or more mental, behavioral, or emotional disorder (classified as any mental illness or AMI) in the past year. It was estimated that 18.3 percent of adolescents aged 12–17 (4.7 million people) and 18.8 percent of adults aged 18 or older (46.5 million people) received mental health services in a specialty setting (2).

Individuals with a SUD may also have other mental disorders and those with mental health disorders may also struggle with substance use (3). Moreover, anxiety, depression, and spiritual distress have been correlated with substance use, where its symptoms are thought to contribute to and result from the complex biopsychosocial process of addiction (4, 5).

Compared to their counterparts who did not have an MDE in the past year, adolescents aged 12–17 with a past year MDE were more likely to use some substances, in the past year or past month (2). In 2021, it was estimated that 935,000 individuals (25.2 percent) among this age group had both an MDE and SUD in the past year.

Mental disorders may contribute to substance use, as individuals with anxiety, depression, or post-traumatic stress disorder (PTSD) may use drugs or alcohol to self-medicate. Other mental health disorders comorbid with SUD can include attention-deficit hyperactivity disorder, bipolar disorder, personality disorders, and schizophrenia. Among adults aged 18 or older, it was estimated that 19.4 million persons (7.6 percent) had comorbidity of AMI and SUD (2).

### 1.3. Mortality associated with substance use

Among persons under the age of 45, accidental drug overdose is the leading cause of death (6). In the United States, there were 106,699 individuals who died from a drug-involved overdose in 2021, with 70,601 deaths involving synthetic opioids other than methadone (primarily fentanyl). Cocaine-involved deaths rose nearly 54% from 15,883 deaths in 2019 to 24,486 deaths in 2021 (7). Excessive alcohol use contributed to more than 140,000 deaths in the United States each year from 2015 through 2019 (8).

### 1.4. Integrative care model

Morbidity and mortality rates are high, biopsychosocial etiologies are complex, and there are mutually sustaining factors between PSU and even subclinical mental distress (3). The treatment of PSU therefore requires comprehensive and individualized treatment plans. The integrative care model at the AIMS Institute includes the principles of prevention, whole-person health (mind–body-spirit), and awareness of whole-systems influence on an individual’s health (9–12). Thus a patient’s mental distress is assessed and treated as one experience, on a spectrum of mortality and morbidity risk, as opposed to siloing one treatment per diagnosis. For example, in-person healthcare delivery for SUD was significantly impacted during the first year of the COVID-19 pandemic. This worsened access to the already limited treatment options for ‘dual diagnoses,’ defined as SUD concurrent with AMI such as depression or anxiety (13). Herein lies the need to explore adjunctive and alternative care models for those with high-risk substance use and comorbid mental distress.

Currently, medication-assisted treatment (MAT) is considered first-line for opioid use disorder. Medications are also approved by the U.S. Food and Drug Administration to treat alcohol use disorder [acamprosate, disulfiram and naltrexone], but overall MAT appears under-utilized in the United States for SUD (14). Depending on the severity of substance use, socioeconomic factors and comorbid mental distress, SUD treatment often involves a time-and resource-intensive combination of medically supervised detoxification and intensive psychotherapy, sometimes with additional group work. While Washington State compares favorably to other states on many health indicators, it has some of the highest rates of mental illness, yet also a severe shortage of mental health professionals including those who can prescribe appropriate medications, those who provide therapy, or providers or facilities that offer both (15).

### 1.5. Ketamine-assisted psychotherapy for problematic substance use

Ketamine-assisted psychotherapy (KAP) utilizes sub-anesthetic doses of the dissociative compound ketamine, along with psychotherapy, to address psycho-emotional distress. This includes depression, anxiety, and spiritual distress, each of which can lead to, co-occur with, or result from substance use disorders. Interestingly, ketamine and acamprosate have in common the mechanism of glutamatergic NMDA receptor antagonism, warranting evaluation of ketamine’s role in SUD treatment alone, as well as treatment of comorbid mental health concerns (16). While still considered an experimental and off-label use of ketamine, KAP also offers several important novel features to the field of SUD treatment: neurobiologically, the promotion of BDNF (brain-derived neurotrophic factor) and neuroplasticity; a supportive set and setting or ‘container’ for mystical experiences and psychospiritual healing; the potential for sustained benefits with few administrations and low risk profile relative to standard of care; and low to no observed risk for habituation to ketamine (17–19).

Ketamine has demonstrated efficacy in prolonging abstinence among individuals with alcohol and heroin use disorders, including after just a single administration in the context of motivational psychotherapy (18–20). This study evaluates the efficacy of KAP in influencing various psychometric scores in individuals with PSU. Symptoms of anxiety were measured by the Generalized Anxiety Disorder-7 (GAD-7) questionnaire. Depression symptoms were measured by the Patient Health Questionnaire-9 (PHQ-9). The National Institute of Health-Healing Experience of All Life Stressors (NIH-HEALS) questionnaire was used to measure psychosocial and spiritual well being. To further contribute to understanding KAP’s utility, specifically for high risk substance use and harm reduction, participant data was compared to population of enrolled study participants who received KAP for any reason during the same time period. A 2022 systematic review of ketamine for mental health and SUD found only three studies of KAP, none of which covered multiple types of substance use or included those at high risk with a preventive approach to treating comorbid conditions (17). To our knowledge this is the only study of ketamine assisted psychotherapy for outpatients at high risk for mixed substance use disorders assessing their comorbid mental distress in comparison to those without PSU.

## 2. Methods

### 2.1. Design and study population

Participants received care at the AIMS (Advanced Integrative Medical Science) Institute, an urban community-based integrative medicine practice in Washington State. This study analyzed a subset of data extracted from two concurrent AIMS Institute prospective outcomes studies: the AIMS Medical Outcomes Study (AMOS) and the AIMS Cancer Outcomes Study (ACOS); (NCT04495790 and NCT04512755, respectively). Both are five-year prospective outcome studies of patient outcomes during and after receiving integrative care at the AIMS Institute (9, 10). Retrospective chart reviews were performed for adolescent and adult patients with established care at AIMS Institute who consented to take part in the AMOS or ACOS studies. Both of the studies have been approved by the Institutional Review Board at Seattle University in Seattle, Washington.

### 2.2. Inclusion and exclusion criteria

Inclusion criteria for the PSU subset were patients who (1) have an active or previous ICD-10 substance use diagnosis per DSM-5 criteria or problematic substance use as defined by the British Columbia Ministry of Health; (2) received at least one KAP experiential session between November 1, 2020-October 31, 2022 at the AIMS Institute; and (3) completed at least one baseline and follow-up psychometric (GAD-7, PHQ-9) or psychosocial and spiritual metrics (NIH-HEALS) questionnaire. Current and former AIMS Institute staff members or volunteer affiliates who received KAP were excluded from this study to reduce the effect of bias upon completing their psychometric assessments.

### 2.3. Measures

In this retrospective chart review, 24 months of data was manually sorted and abstracted from two electronic health record (EHR) systems for patients who received ketamine-assisted psychotherapy at the AIMS Institute in Seattle, Washington within the time frame of November 1, 2020 to October 31, 2022. One platform serves as the primary EHR, storing patient data, demographics, and associated diagnoses. A second EHR platform tracks various psychometric scores over time; this system regularly prompts patients to submit responses every 90 days to self-report questionnaires. Questionnaires utilized were the PHQ-9 for severity of depression symptoms, the GAD-7 for severity of anxiety symptoms, and the National Institute of Health – Healing Experiences from All Life Stressors scale (NIH-HEALS), a validated 35-item scale to assess the patient’s state of psychosocial and spiritual elements of health resilience and well being. Improvement is measured with decreased PHQ-9 and GAD-7 ratings and with increased NIH-HEALS ratings.

The following data was collected: basic demographics; dates of KAP sessions; type of ketamine treatment received; diagnosis of AMI, SUD, or conditions that may benefit from KAP (e.g., chronic pain, insomnia); type of substance used; psychiatric medications 1 month leading up to the first KAP session; pre-medications utilized on day of KAP; adverse side effects; and various questionnaire scores (ACE, Resilience, GAD-7, PHQ-9, NIH-HEALS). Due to the low volume of data of documented SUD, clinician recall was additionally used to identify patients with histories of SUD or active PSU of any severity.

Baseline GAD-7, PHQ-9, and NIH-HEALS scores were established using the most recent questionnaires that preceded a participant’s first KAP session. Changes were calculated by comparing the difference between the baseline questionnaire score and the last post-KAP questionnaire score completed. Statistical analyses were performed on paired data. In assessing the relationship between time elapsed since KAP (or prior to KAP) to psychometric outcomes, the number of days prior to or after KAP were calculated for each participant and correlated to GAD-7-PHQ-9, and NIH-HEALS scores.

The participant results were compared to the entire study-enrolled population of KAP recipients at AIMS during the same two-year time period. In this comparison, the participant group is termed the participant group is termed PSU Subpopulation.

### 2.4. Statistical analysis

Microsoft Excel software was used for statistical analysis. A majority of the data was analyzed utilizing descriptive statistics (counts, percentages, means, and standard deviation). Due to ssmall sample size of the PSU Subpopulation (*n* = 18), Wilcoxon rank-sum tests were performed between baseline and final metric scores with an established significance level of *ɑ* < 0.05. For the larger KAP population, paired *t*-tests examined differences between baseline and final metric scores with an established significance level of *p* < 0.05. An independent samples *t*-test compared average changes between the PSU Subpopulation and KAP Population. Linear regression analysis was performed on psychometric scores. The correlation between receiving KAP and psychometric score changes was analyzed with Pearson correlation analysis.

## 3. Results

There were 307 patients who received at least one KAP session between November 1, 2020 and October 31, 2022. After applying exclusions, a total of 291 charts were reviewed and 230 consents were confirmed. The percentage of IRB-approved study enrollment specific to KAP treatments for this time frame was 79.04%. There were 30 patients identified to have problematic substance use. After applying inclusions, a total of 18 patients completed at least one KAP session and at least one baseline and post-KAP psychometric questionnaire, which comprises the participant population. Baseline psychometrics were completed on average 65 days prior to KAP, and follow up measures were completed on average 59 days after KAP.

### 3.1. Demographics

Participants ranged in age from 16 to 67 years. The mean participant age at the time of their first ketamine session was 39.56 years. A majority of participants were in their 30s (*n* = 7; mode = 48 years; median = 38 years). Participants identified their gender as follows: female (*n* = 11), male (*n* = 6) and genderqueer (*n* = 1).

Within the context of problematic substance use, a majority of participants used alcohol (*n* = 10, 55.56%), followed by nicotine (*n* = 5, 27.78%), and opioids (*n* = 2, 11.11%). Other substances used include benzodiazepines, cocaine, heroin, zolpidem, and an unknown psychedelic substance (each with *n* = 1, 5.56%). Most participants reported using one substance (*n* = 15, 83.3%) to their AIMS provider. Two participants (11.11%) were identified to have used two substances. One participant (5.56%) was identified to have used three substances.

The average Adverse Childhood Experiences (ACE) and Resilience scores were 3.89 ± 2.47 and 10.17 ± 2.96, respectively. The number of KAP experiential sessions attended by each participant ranged from 1 to 6 sessions, with a median of 3 sessions. Table 1 outlines each participant’s characteristics.

**Table 1:** Participant demographics, substances used, Adverse childhood experiences (ACE) and resilience scores, and number of KAP sessions completed.

Study ID	Age (years) at time of first KAP	Gender	Substance(s) used	ACE score	Resilience score	Number of KAP sessions
1	18	Genderqueer	Alcohol	5	7	2
2	67	Male	Alcohol	3	6	3
3	33	Female	Nicotine	9	12	1
4	31	Female	Nicotine	5	11	3
5	38	Male	Alcohol, cocaine, benzodiazepines	4	12	1
6	35	Female	Alcohol	3	13	4
7	44	Female	Alcohol	5	10	3
8	60	Female	Alcohol	2	2	3
9	48	Female	Alcohol	1	10	1
10	37	Female	Opioids, zolpidem	2	13	3
11	38	Female	Alcohol	2	9	6
12	48	Male	Opioids	0	13	3
13	23	Female	Nicotine	3	10	4
14	16	Male	Psychoactive substance unknown	1	14	2
15	34	Male	Alcohol, nicotine	5	11	1
16	51	Male	Alcohol	5	8	4
17	48	Female	Nicotine	8	11	3
18	43	Female	Heroin	7	11	4

### 3.2. Comorbidities

At the time of the first KAP treatment, participants ranged from two to eight comorbid conditions with a mode of four conditions. The most common comorbid conditions were a type of depression (*n* = 14, 77.8%), a type of anxiety (*n* = 10, 55.6%), and post-traumatic stress disorder (*n* = 10, 55.6%). Conditions such as insomnia, chronic pain, traumatic brain injury, seizure disorders, and migraines were also noted. Table 2 outlines the PSU population’s comorbid conditions.

**Table 2:** Comorbid diagnoses in participant population (*n* = 18) during the time of first KAP treatment.

Diagnosis	*n*	Diagnosis	*n*
Adjustment disorder, any type	7	Mania	1
Anxiety, any type	10	Medication induced psychosis	1
Attention deficit hyperactivity disorder	4	Migraines	1
Bipolar disorder	2	Obsessive compulsive disorder	1
Chronic pain	5	Post-traumatic stress disorder	10
Depression, other types	12	Stress	3
Depression, post-partum	2	Suicide (ideations, attempts)	4
Eating disorder	1	Trauma/abuse history	6
Epilepsy	1	Traumatic brain injury	1
Insomnia	4		

### 3.3. Medications

#### 3.3.1. Medications utilized 1 month leading up to the first KAP session

There were 14 different classes of psychotropic medications concurrently used by study participants. The most common concurrent medications were serotonin-norepinephrine reuptake inhibitors, anticonvulsants, and the serotonin antagonist-reuptake inhibitor trazodone as a sleep aid (each at *n* = 4, 22%). Medication classes and rates of concurrent use leading up to participants’ first KAP session are listed in Table 3.

**Table 3:** Concurrent psychotropic medications in participant population (*n* = 18) at the time of their first KAP experience, from most common to least common medication class.

Medication class	*n*	Medication class	*n*
SNRI (venlafaxine, desvenlafaxine)	4	Alpha agonist (clonidine)	2
SARI (trazodone)	4	Antipsychotic (olanzapine, aripiprazole)	2
Anticonvulsant (gabapentin, lacosamide)	4	Beta blocker (propranolol)	2
Amphetamines (Adderall, Vyvanse)	3	Opioid agonist (methadone, hydrocodone)	2
NDRI (bupropion)	3	Anxiolytic (buspirone)	1
SSRI (escitalopram)	3	Opioid partial agonist (buprenorphine)	1
Benzodiazepine (clonazepam, lorazepam)	3	Muscle relaxant (cyclobenzaprine)	1

#### 3.3.2. KAP pre-medication

There were 16 (88.9%) participants who utilized ondansetron prior to their KAP session. Other pre-medications utilized were meclizine (*n* = 7, 38.9%) and propranolol (*n* = 1, 5.6%). Each participant utilized no more than two pre-medications at the time of their KAP session.

### 3.4. Adverse side effects

The most common reported adverse reactions to ketamine were nausea and dizziness (each at *n* = 4, 22%), followed by fatigue (*n* = 2, 11%). More uncommon adverse effects were vomiting, dry mouth, and slower movements (each at *n* = 1, 5.6%).

### 3.5. Baseline and post-KAP session PHQ-9, GAD-7, and NIH-HEALS

**Figure 1:**
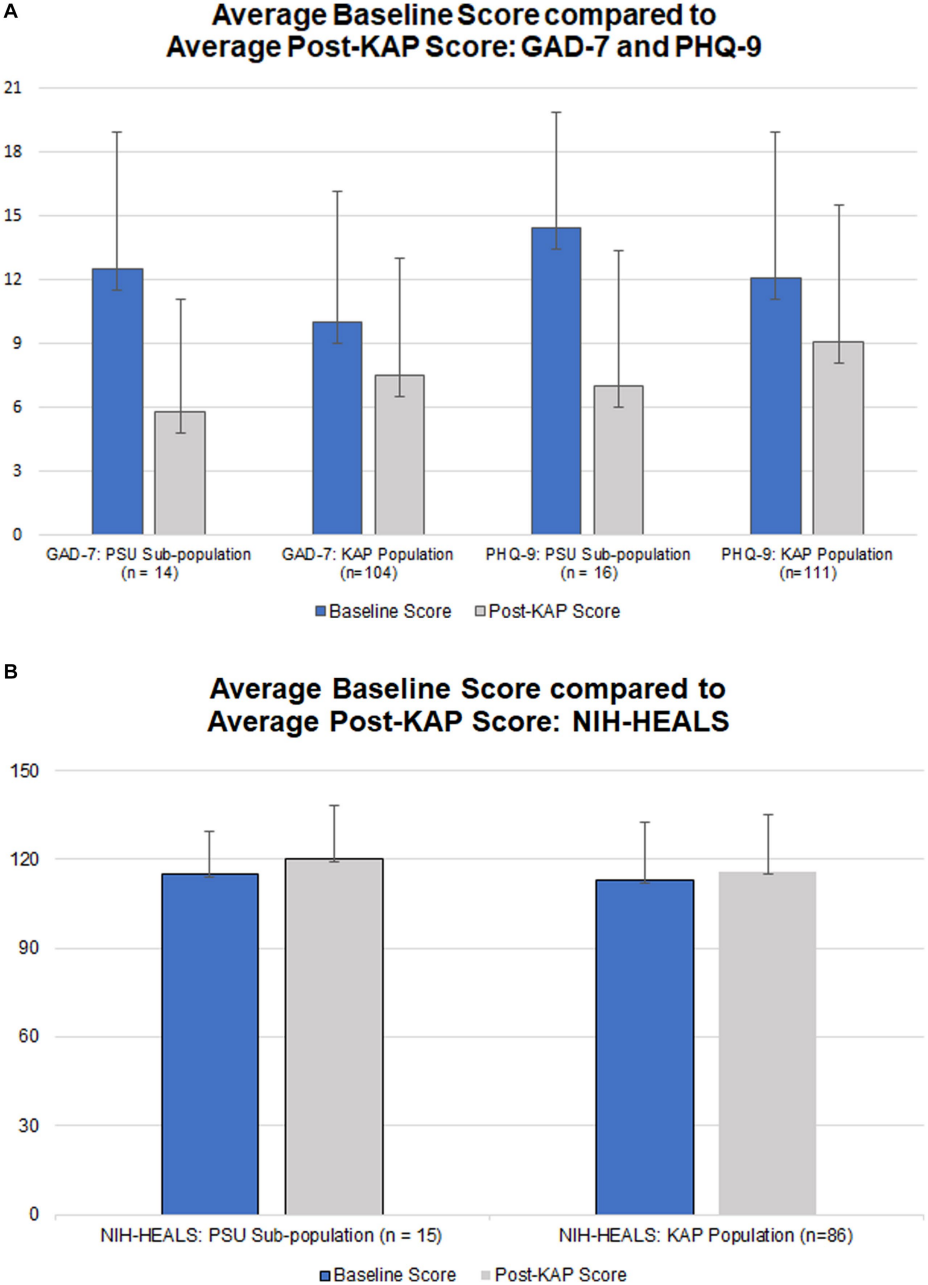
**(A)** Average baseline GAD-7 and PHQ-9 scores (blue) and respective average post-KAP scores (gray) for the problematic substance use population (‘PSU Subpopulation’) in comparison to the greater population of enrolled KAP recipients; score range 0–21 for GAD-7 and 0–27 for PHQ-9, with lower scores indicating fewer symptoms. **(B)** Average baseline NIH-HEALS score (blue) compared to average post-KAP scores (gray) for the problematic substance use population (‘PSU Subpopulation’) in comparison to the greater population of enrolled KAP recipients; score; score range 35–175, with higher scores indicating improved response to challenging life events.

Average baseline score, last completed score, change in scores, and comparison of average changes for corresponding questionnaires and populations are outlined in Table 4. The scores for the problematic substance use participant population (named ‘PSU Subpopulation’) can be compared against the score for the KAP population. Figures 1A,B illustrate the average score changes when compared to baseline for both the participant population and the KAP population.

**Table 4:** Average baseline score, last completed score, change in scores, and comparison of average changes for corresponding questionnaires and populations.

Questionnaire and population	*n*	Average baseline score	Average last completed score	Average change in scores	Comparison of average changes (*p* < 0.05)
GAD-7; PSU Subpopulation (*n* = 18)	14	12.5 ± 6.48	5.79 ± 5.29	−6.71 ± 9.15	0.0219 (95% C.I. 0.63 to 7.89)
GAD-7; KAP Population (*n* = 230)	104	9.99 ± 6.17	7.54 ± 5.46	−2.45 ± 6.01	
PHQ-9; PSU Subpopulation (*n* = 18)	16	14.44 ± 5.42	7 ± 6.36	−7.44 ± 5.42	0.0062 (95% C.I. 1.28 to 7.56)
PHQ-9; KAP Population (*n* = 230)	111	12.08 ± 6.85	9.06 ± 6.47	−3.02 ± 6.01	
NIH-HEALS; PSU Subpopulation (*n* = 18)	15	115.07 ± 14.62	120.2 ± 17.87	5.13 ± 13.64	0.5197 (95% C.I. 8.96–4.56)
NIH-HEALS; KAP Population (*n* = 230)	86	112.97 ± 19.45	115.9 ± 19	2.93 ± 11.91	

Participants who completed at least one post-KAP GAD-7 questionnaire (*n*=14, 77.78%) had baseline GAD-7 scores ranging from 1 to 21 points. After at least one KAP experiential session, scores ranged from 0 to 18 points. The score range for GAD-7 is 0–21 with higher scores indicating worsening severity of anxiety symptoms. The Wilcoxon rank-sum test calculated *U* = 42. This rejects the null hypothesis (ɑ = 0.05 of 55). The enrolled KAP population paired t-test result for GAD-7 scores was statistically significant at *p* = 6.64 x 10^−5^ (*n* = 104).

There were 88.89% (*n* = 16) of participants who completed at least one post-KAP PHQ-9 questionnaire. Baseline PHQ-9 scores ranged from 4 to 23 points. After at least one KAP experiential session, scores ranged from 0 to 17 points. The score range for PHQ-9 is 0–27 with higher scores indicating worsening severity of depression symptoms. The Wilcoxon rank-sum test calculated *U* = 52, rejecting the null hypothesis (*ɑ* = 0.05 of 75). The enrolled KAP population paired t-test result for PHQ-9 scores was statistically significant at *p* = 6.25 x  10^−7^ (*n* = 111).

There were 83.33% (*n* = 15) of participants who completed at least one post-KAP NIH-HEALS questionnaire. Baseline NIH-HEALS scores ranged from 94 to 146 points. After at least one KAP session, scores ranged from 79 to 146 points. The score range for NIH-HEALS is 35–175, with higher scores indicating greater positive transformation in response to challenging life events. The Wilcoxon rank-sum test calculated *U* = 139. This did not reject the null hypothesis (*ɑ* = 0.05 of 64). The enrolled KAP population paired t-test result for NIH-HEALS scores was statistically significant at *p* = 0.037 (*n* = 85).

**Figure 2:**
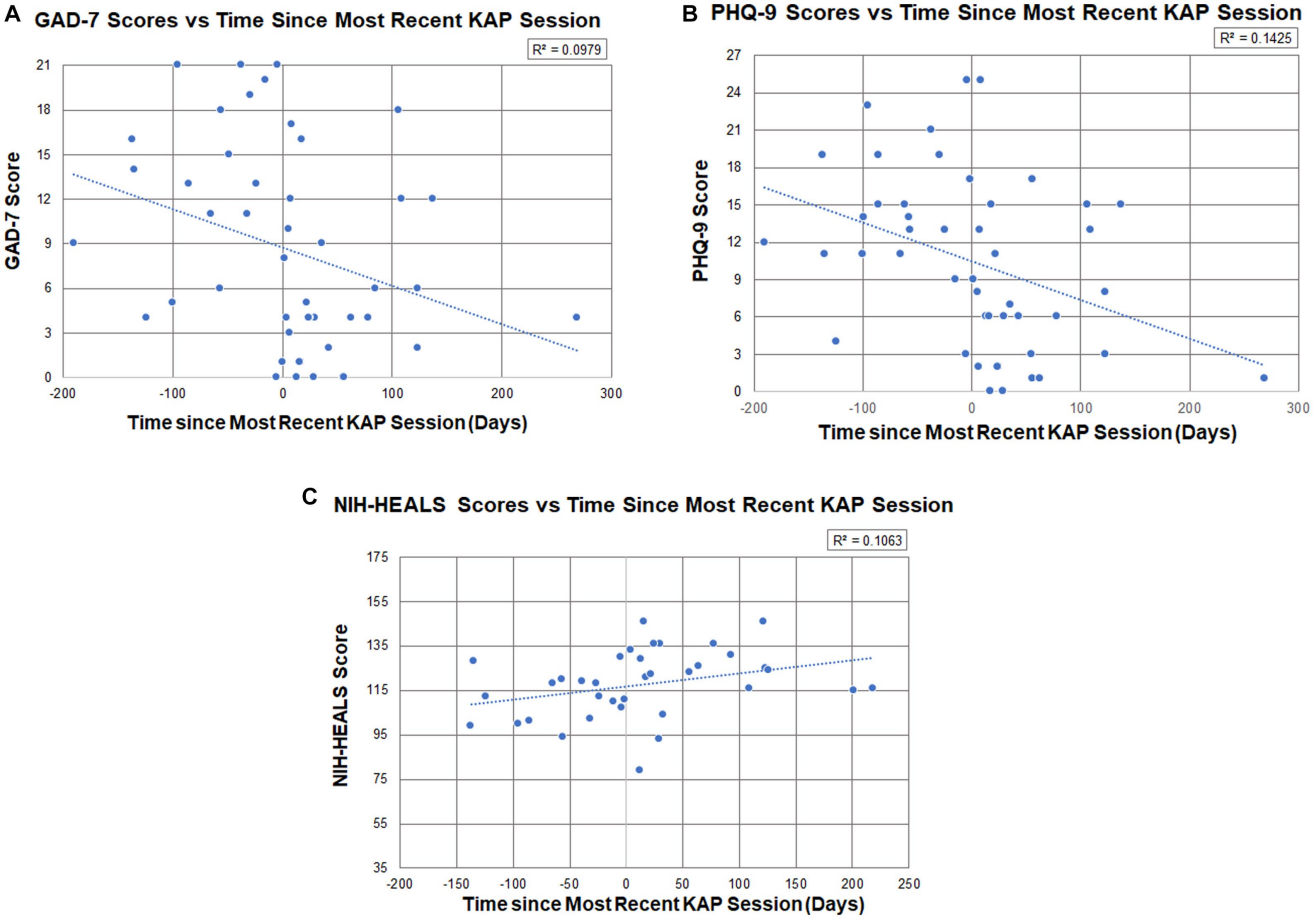
**(A)** Participant GAD-7 scores (*n* = 14, total measurements = 47) in relation to the number of days prior to or after a KAP session (*R*^2^ = 0.098), where days were measured from the date of the soonest upcoming KAP session (first score establishes baseline) to their most recent KAP session (post-KAP score). **(B)** Participant PHQ-9 scores (*n* = 16, total measurements = 47) in relation to the number of days prior to or after a KAP session (*R*^2^ = 0.143), where days were measured from the date of the soonest upcoming ketamine session (first score establishes baseline) to their most recent ketamine administration (post-KAP score). **(C)** Participant NIH-HEALS scores (*n* = 15, total measurements = 36) in relation to the number of days prior to or after a KAP session (*R*^2^ = 0.106), where days were measured from the date of the soonest upcoming KAP session (first score establishes baseline) to their most recent ketamine administration (post-KAP score).

Participants’ psychometric scores and calculated changes between the baseline score and the last post-KAP questionnaire score completed are described in Table 5. Figures 2A–C illustrate respective psychometric scores plotted by the number of days prior to or after a KAP session. In terms of correlations, the models illustrate that there is partial predictability to outcomes with medium effects on post-KAP psychometric scores. The *R*^2^ values for GAD-7, PHQ-9, and NIH-HEALS were calculated as 0.098, 0.143, and 0.106, respectively.

**Table 5:** Participant’s baseline metric score, post-KAP score, and calculated change between the baseline questionnaire score and the last post-KAP questionnaire score completed.

Study ID	GAD-7			PHQ-9			NIH-HEALS		
	Baseline score	Post-KAP score	Change to score	Baseline score	Post-KAP score	Change to score	Baseline score	Post-KAP score	Change to score
1	9	10	1	12	9	−3	105	No data	No data
2	19	18	−1	19	15	−4	92	No data	No data
3	18	0	−18	13	3	−10	94	79	−15
4	13	12	−1	13	15	2	112	115	3
5	1	No data	No data	8	No data	No data	140	134	−6
6	11	2	−9	14	3	−11	118	146	28
7	21	No data	No data	23	17	−6	100	131	31
8	6	4	−2	14	6	−8	120	136	16
9	14	4	−10	11	1	−10	128	130	2
10	14	No data	No data	21	16	−5	90	No data	No data
11	21	1	−20	21	0	−21	118	133	15
12	1	4	3	6	2	−4	146	136	−10
13	16	12	−4	19	13	−6	99	101	2
14	1	4	3	17	1	−16	111	126	15
15	7	No data	No data	6	No data	No data	109	104	−5
16	11	0	−11	4	0	−4	102	112	10
17	20	4	−16	9	2	−7	110	104	−6
18	15	6	−9	15	9	−6	119	116	−3

## 4. Discussion

There was statistically significant evidence to show that having at least one ketamine-assisted psychotherapy session improved symptoms of anxiety and depression in participants with PSU. This further supports recent reviews of literature indicating that ketamine may have clinical applications in the treatment of refractory anxiety disorders and continues to improve depressive symptoms in those with major depressive disorder and/or bipolar disorder (21–23). Ketamine also has the added benefit of being generally well tolerated having a limited side effect profile (21, 22). Participants in this study reported nausea and dizziness as the most common adverse effects and most (*n* = 16) received pre-medication to mitigate these effects.

Substance use has been correlated as a coping method for stress and emotions (3). During the earlier months of the COVID-19 pandemic (June 2020), the Center for Disease Control and Prevention noted that 40% of adults in the United States reported struggling with mental health or substance use with 13% starting or increasing substance use (24). These findings suggest that KAP may be helpful in mitigating the risk of problematic substance use by relieving some symptoms of depression and anxiety. This represents an integrative treatment approach by reducing the impact of sustaining factors of high risk substance use, preventing progression to frank SUD, and addressing mind and spirit.

There is not sufficient evidence to suggest statistically significant improvements to NIH-HEALS scores after receiving at least one KAP treatment. However, among one-third (*n* = 6) of respondents, there was a decrease in NIH-HEALS scores after KAP. This feature of the data and possible explanations are discussed in the Supplementary material (6.2.1).

The KAP population of enrolled subjects (*n* = 230) was compared to the PSU group to gain an understanding of the unique psychometric features and responses to KAP among those with PSU. When comparing the PSU group to the enrolled KAP population, the difference in changes between average baseline scores and last documented scores were statistically significant for GAD-7 (*p* = 0.0219) and PHQ-9 (*p* = 0.0062) and not statistically significant for NIH-HEALS (*p* = 0.5197). Those with PSU in particular may benefit from KAP for anxiety and depression, more so than a general population of patients receiving KAP for any indication.

### 4.1. Improvement to at least one domain: GAD-7 and PHQ-9 psychometric scores

A majority of participants with PSU had comorbid anxiety (*n* = 10) and/or depression (*n* = 12). Overall, data showed improvements to psychometric scores after having at least one KAP session. The average reductions in GAD-7 scores by 6.71 ± 9.15 points and PHQ-9 scores by 7.44 ± 5.42 points from baseline are statistically significant. Additionally, Pearson’s correlation indicates that there is practical significance of having at least one KAP session influence psychometric scores with medium effects (GAD-7: *R*^2^ = 0.098; PHQ-9: *R*^2^ = 0.143). GAD-7 scores show reductions to anxiety symptoms, where the average baseline scores in the moderate range (10–14) shifted to the mild range (5–9) post-KAP. The same trend was seen with depression symptoms on the PHQ-9, where the average baseline scores changed from the moderate range (10–14) to the mild range (5–9) post-KAP.

All participants who completed follow-up psychometric testing demonstrated improvement in at least one domain. For example, when referring to Table 5, participants #1, #12, and #14 had increases to their GAD-7 scores, implying worsened anxiety symptoms. However, these same participants also had decreases in PHQ-9 scores, implying improved depression symptoms.

### 4.2. Best outcome with greater number of KAP sessions in a shorter time interval

Participants experienced more improvements to psychometric scores when they also completed more KAP sessions in a shorter time interval. One participant (#11) who completed the greatest number of in-office KAP sessions during the study time period (6 sessions), also participated in a KAP session at the most frequent rate: every other week. This participant had the highest improvements to GAD-7 and PHQ-9 scores. These outcomes corroborate a 2022 systematic literature review, which found that higher doses of ketamine, more frequent KAP sessions, and longer durations of psychotherapy increase the efficacy and durability of improvements in conditions such as depression, anxiety, and SUD (25). However, there are participants who experienced benefit after fewer KAP sessions. Two participants (#3, #9) reported significant reductions in anxiety and depression after only one session and one participant’s (#14) depression score significantly decreased after two sessions (Tables 1, 5).

### 4.3. Durability of benefit

Little is known about ketamine’s long-term efficacy in addressing anxiety and depression(21, 23). Trends in PHQ-9, GAD-7 and NIH-HEALS show sustained improvement since the most recent ketamine administration. Psychometric responses were gathered by self-report. As such, there is a wide variety of time intervals between KAP sessions and repeat questionnaire completion (Figures 2A–C). Self-reports up to 269 days (8.8 months) after KAP, with an average of 59 days between responses, indicate that the statistically significant changes to anxiety and depression scores are not attributable to only the acute or subacute effects of ketamine, and may represent a durability of effect or new mutually sustaining protective features in a population with PSU. Given the months-years of development and complex etiology of PSU, investigating appropriate interventions with persisting effects without repeated administrations is valuable (26). Outcome intervals in integrative medicine research frameworks are often months long to reflect durable change in foundations of health, with the Bravewell Collaborative’s National Integrative Medicine Database (PRIMIER) collection of self-report data at three 2 month intervals, followed by three 6-month intervals (27). The data captured in our subset analysis, while the sample size and *R*^2^ values are modest, are preliminary suggestions of durability of benefit in depression, anxiety and psycho-spiritual health for those with PSU. Further commentary on the trend of improved psychometric measures as a patient’s ketamine experience was approaching is provided in Supplementary material (6.3.1).

### 4.4. Limitations

A limitation of this study includes possible non-response bias since data collected is dependent on patients consistently submitting self-report measures. The standard practice at the AIMS Institute is to regularly collect PHQ-9, GAD-7, and NIH-HEALS every 90 days after establishing baseline; however, these psychometric survey responses vary widely in their proximity to KAP experiential sessions (and integration visits). Minimizing non-response and regularizing psychometric intervals was attempted by sending out automated questionnaire reminders via the secondary EHR platform, collecting psychometrics through the primary EHR platform, and handing out paper questionnaires where necessary. Despite this, response rates remain low (50–56%). Participants who chose not to repeat psychometric scores may have done so for a number of reasons, including a poor or neutral outcome. This also introduces selection bias to the pool of available results.

Another limitation is that there is no consistent data collected to definitively address whether KAP is effective at reducing the frequency of PSU. As data was collected retrospectively for those with a history of high risk substance use, we did not consistently collect substance use data prior to and after KAP. This study did not include a matched control group, nor are we able to control for other various interventions for psycho-emotional health utilized by participants during the study time period, given the outpatient clinical context of the study. Though there are statistically relevant findings demonstrating improvements to anxiety and depression scores with KAP to a medium effect, a small sample size limits the power of results reported here. Variations in the number of KAP sessions patients received between baseline and psychometric follow up measurements limit our observations regarding KAP for PSU.

### 4.5. Implications for future research

With a greater sample size, future research could assess differences among high-risk substance use in those with and without clinical depression or anxiety and provide a matched-control by including those with PSU who receive integrative care without KAP.

#### 4.5.1. Measuring severity and duration of substance use before and after KAP

While perhaps decreased symptoms of anxiety and depression after KAP may result in lesser PSU as a means to cope, additional studies could clarify this by gathering substance use data. Studies that quantitatively evaluate the frequency and severity of substance use including prevention of relapse are necessary for a comprehensive analysis of KAP’s efficacy for treating active SUD.

#### 4.5.2. NIH-HEALS correlations

Further investigation is needed to corroborate or explain the simultaneous improvement in anxiety and depression scores despite decreased psychosocial and spiritual scores in 33% of NIH-HEALS respondents. Preliminary results from our small sample size reveal that of the patients whose NIH-HEALS scores declined (*n* = 5), all had been diagnosed with post-traumatic stress, all had been diagnosed with post-traumatic stress, including three diagnoses of post-traumatic stress disorder (PTSD; 50%). However, two participants who reported the most significant gains in NIH-HEALS scores also carried PTSD diagnoses. Sixty percent of patients who reported NIH-HEALS improvements by greater than 15 points (*n* = 3 of 5) carried diagnoses of suicidal ideation or sequelae of a suicide attempt. Further studies with greater sample sizes and standardized time windows for NIH-HEALS measurement are warranted to evaluate the psychosocial and spiritual effects of KAP for those with PTSD, chronic or acute suicidality and PSU. See Supplementary material for further commentary (6.2.1).

### 4.6. Implications for clinical practice

Ketamine-assisted psychotherapy should be considered early in the development of an integrative and individualized treatment plan for patients with known comorbid PSU of any kind and symptoms of anxiety or depression. Thorough screening for symptoms of anxiety and depression should be performed for any patient-or clinician-identified problematic substance use. Practices for including KAP in an integrative model could include thorough assessment of comorbid PTSD and the presence or history of suicidality as these symptoms could influence the psychosocial and spiritual effects of KAP.

Additional strategies may be necessary for increasing the rate of follow-up psychometric reporting for outpatient research studies in integrative mental health. For this clinic, only around 50–56% of the participant population completed at least one repeat psychometric questionnaire. Barriers to collecting this data may exist in the clerical systems and research protocols at this institute and in other urban outpatient community-based clinics. Additional patient reminders during post-ketamine care (including integration sessions) or including clinician-administered psychometrics may close some gaps in data collection.

## 5. Conclusion

The data evaluated herein is a subpopulation of the ACOS and AMOS prospective outcomes study of individuals who have problematic substance use (PSU) and who completed at least one ketamine assisted psychotherapy (KAP) session at the AIMS Institute between November 1, 2020 and October 31, 2022 (*n* = 18, median number of ketamine sessions = 3). Comparing baseline to post-KAP psychometric scores, statistically and clinically significant findings demonstrate that KAP provided in an urban outpatient integrative clinic facilitates improvements in anxiety and depression symptoms in individuals who also have PSU.

Additional analysis suggests that patients with PSU and comorbid anxiety and depression symptoms benefit significantly more, on average, than a population of patients receiving KAP for any indication. Further studies are warranted to determine whether greater clinical improvement in PSU results from early identification and intervention of anxiety and depression, and to further elucidate why the population with PSU experiences greater benefits on average than the general KAP patient population. Correlation should be done between improvement in psychometrics and whether participants experience decreased substance cravings, decreased risky use or the use of other protective coping strategies. In terms of KAP facilitating greater positive transformation in response to challenging life events (evaluated by NIH-HEALS), there is no statistically significant finding to this correlation. While there was a general improvement in NIH-HEALS scores, it is important to note that scores declined in one-third of the study population. This may be explained by items of the NIH-HEALS measuring aspects of psycho-socio-spiritual health that may have been directly impacted by the COVID-19 pandemic, such as access to social support, or as a result of KAP shifting psycho-spiritual beliefs and experiences.

## Data availability statement

The raw data supporting the conclusions of this article will be made available by the authors, without undue reservation.

## Ethics statement

The studies involving human participants were reviewed and approved by the Institutional Review Board at Seattle University in Seattle, Washington. Written informed consent to participate in this study was provided by the participants or their legal guardian/next of kin.

## Author contributions

EW designed and planned the study, conducted the data collection, abstraction, performed the data analyses, and drafted the manuscript. TE designed and planned the study, planned and conducted the data collection, abstraction, performed the data analyses, and drafted the manuscript. MJ and AS planned and conducted the data collection, abstraction and drafted portions of the manuscript. SA conceived, designed, and planned the study and revised the manuscript. All authors agree to be accountable for the content of the work and contributed to the article and approved the submitted version.

## Conflict of interest

EW was employed by the AIMS Institute.

The remaining authors declare that the research was conducted in the absence of any commercial or financial relationships that could be construed as a potential conflict of interest.

## Publisher’s note

All claims expressed in this article are solely those of the authors and do not necessarily represent those of their affiliated organizations, or those of the publisher, the editors and the reviewers. Any product that may be evaluated in this article, or claim that may be made by its manufacturer, is not guaranteed or endorsed by the publisher.

## References

[1] Every Door is the Right door: A British Columbia planning framework to address problematic substance use and addiction. (2004). British Columbia Ministry of Health services. Available at: https://www.uvic.ca/research/centres/cisur/assets/docs/every-door.pdf

[2] US Department of Health and Human Services: Substance Abuse and Mental Health Services Administration: Center for Behavioral Health Statistics and Quality. Key Substance Use and Mental Health Indicators in the United States: Results from the 2021 National Survey on drug use and health. HHS Publ no PEP22-07-01-005 NSDUH Ser H-57. (2022). Available at: https://www.samhsa.gov/data/sites/default/files/reports/rpt39443/2021NSDUHFFRRev010323.pdf.

[3] NIMH Resource Center. Substance use and co-occurring mental disorders. National Institute of Mental Health (NIMH). Available at: https://www.nimh.nih.gov/health/topics/substance-use-and-mental-health (Accessed April 18, 2023).

[4] Worrell SD Gould TJ. Therapeutic potential of ketamine for alcohol use disorder. Neurosci Biobehav Rev. (2021) 126:573–89. doi: 10.1016/j.neubiorev.2021.05.006 33989669

[5] Lucchetti G Koenig HG Lucchetti ALG. Spirituality, religiousness, and mental health: a review of the current scientific evidence. World J Clin Cases. (2021) 9:7620–31. doi: 10.12998/wjcc.v9.i26.7620 34621814 PMC8462234

[6] NCDAS: Substance abuse and addiction statistics. (2023). NCDAS. Available at: https://drugabusestatistics.org/ (Accessed April 17, 2023).

[7] Abuse NI on D. Drug overdose death rates. National Institute on Drug Abuse. (2023). Available at: http://nida.nih.gov/research-topics/trends-statistics/overdose-death-rates (Accessed April 18, 2023).

[8] CDC. Alcohol-related deaths. Centers for Disease Control and Prevention. (2022). Available at: https://www.cdc.gov/alcohol/features/excessive-alcohol-deaths.html (Accessed April 18, 2023).

[9] Advanced integrative medical science institute. AIMS Medical Outcomes Study clinicaltrialsgov. (2023). Available at: https://clinicaltrials.gov/ct2/show/NCT04512755 Accessed February 5, 2023.

[10] Advanced integrative medical science institute. Advanced integrative oncology treatment for adult and pediatric patients with Cancer: a prospective outcomes study. clinicaltrials.gov. (2023). Available at: https://clinicaltrials.gov/ct2/show/NCT04495790 Accessed February 5, 2023.

[11] Integrative medicine defined | ABPS | physician board certification. American Board of Physician Specialties. Available at: https://www.abpsus.org/integrative-medicine-defined/ (Accessed April 17, 2023).

[12] Association of Accredited Naturopathic Medial Colleges. The six principles of naturopathic medicine. AANMC. Available at: https://aanmc.org/6-principles/ (Accessed April 17, 2023).

[13] Trends in visits to substance use disorder treatment facilities in 2020 - PMC. Accessed February 6, 2023. Available at: https://www.ncbi.nlm.nih.gov/pmc/articles/PMC8217724/10.1016/j.jsat.2021.108462 PMC8217724 34134879

[14] Fischler PV Soyka M Seifritz E Mutschler J. Off-label and investigational drugs in the treatment of alcohol use disorder: a critical review. Front Pharmacol. (2022) 13:927703. doi: 10.3389/fphar.2022.927703 36263121 PMC9574013

[15] Mental-health needs have Washington in a state of crisis. UW Magazine — University of Washington Magazine. Available at: https://magazine.washington.edu/feature/mental-health-needs-have-washington-in-a-state-of-crisis/ (Accessed January 21, 2023).

[16] Rosenbaum SB Gupta V Patel V Palacios JL. Ketamine. StatPearls: StatPearls Publishing (2017). Available at: https://europepmc.org/article/nbk/nbk470357#free-full-text (Accessed April 17, 2023). 29262083

[17] Walsh Z Mollaahmetoglu OM Rootman J Lawn W Keeler J Marsh B. Ketamine for the treatment of mental health and substance use disorders: comprehensive systematic review. BJPsych Open. (2021) 8:e19. doi: 10.1192/bjo.2021.1061 35048815 PMC8715255

[18] Grabski M McAndrew A Lawn W Marsh B Raymen L Stevens T. Adjunctive ketamine with relapse prevention-based psychological therapy in the treatment of alcohol use disorder. Am J Psychiatry. (2022) 179:152–62. doi: 10.1176/appi.ajp.2021.21030277 35012326

[19] Ivan Ezquerra-Romano I Lawn W Krupitsky E Morgan CJA. Ketamine for the treatment of addiction: evidence and potential mechanisms. Neuropharmacology. (2018) 142:72–82. doi: 10.1016/j.neuropharm.2018.01.017 29339294

[20] Dakwar E Levin F Hart CL Basaraba C Choi J Pavlicova M. A single ketamine infusion combined with motivational enhancement therapy for alcohol use disorder: a randomized midazolam-controlled pilot trial. Am J Psychiatry. (2020) 177:125–33. doi: 10.1176/appi.ajp.2019.19070684 31786934

[21] Banov MD Young JR Dunn T Szabo ST. Efficacy and safety of ketamine in the management of anxiety and anxiety spectrum disorders: a review of the literature. CNS Spectr. (2020) 25:331–42. doi: 10.1017/S1092852919001238 31339086

[22] Yavi M Lee H Henter ID Park LT Zarate CA. Ketamine treatment for depression: a review. Discov Ment Health. (2022) 2:9. doi: 10.1007/s44192-022-00012-3 35509843 PMC9010394

[23] Corriger A Pickering G. Ketamine and depression: a narrative review. Drug Des Devel Ther. (2019) 13:3051–67. doi: 10.2147/DDDT.S221437 PMC6717708 31695324

[24] Czeisler MÉ Lane RI Petrosky E Wiley JF Christensen A Njai R. Mental Health, Substance Use, and Suicidal Ideation During the COVID-19 Pandemic — United States, June 24–30, 2020. MMWR Morb Mortal Wkly Rep. (2020) 69:1049–1057. doi: 10.15585/mmwr.mm6932a1 32790653 PMC7440121

[25] Drozdz SJ Goel A McGarr MW Katz J Ritvo P Mattina GF. Ketamine assisted psychotherapy: a systematic narrative review of the literature. J Pain Res. (2022) 15:1691–706. doi: 10.2147/JPR.S360733 35734507 PMC9207256

[26] Common comorbidities with substance use disorders research report. National Institutes on drug abuse (US). (2020). Available at: http://www.ncbi.nlm.nih.gov/books/NBK571451/ (Accessed April 17, 2023). 34185444

[27] Abrams D Dolor R Dusek J Horrigan B Kligler B McKee D. PRIMIER: A national integrative medicine database. The Bravewell Collective (2015). https://aimforwellbeing.org/wp-content/uploads/2021/08/PRIMIER-Rpt.pdf

