# Supplementary material for: Reductions in anxiety and depression symptoms in a subset of outpatients with problematic substance use who received ketamine-assisted psychotherapy: a two-year retrospective chart review
Source: Front Psychiatry. 2023 Aug 30;14:1160442. doi: 10.3389/fpsyt.2023.1160442 (PMC10498542; doi:10.3389/fpsyt.2023.1160442)
Supplement: Supplementary file 1 [file Data_Sheet_1.pdf]

Reductions in anxiety and depression symptoms in a subset of outpatients with problematic substance use who received ketamine-assisted psychotherapy: a two-year retrospective chart review.

Whinkin, Emily[1*]; Eparwa, Therry Rose J. [1, 2]; Julseth, Michelle C.[2]; Schneider, Andrea[2]; Aggarwal, Sunil K.[1]

[1] Advanced Integrative Medical Sciences Institute, Seattle, WA, United States of America

[2] Seattle University, College of Nursing, Seattle, WA, United States of America

*** Author Correspondence:**
Emily Whinkin
ewhinkin@aimsinstitute.net

Keywords: ketamine, ketamine-assisted psychotherapy, substance use disorders, anxiety, depression, psychometrics, spiritual distress

6. Supplementary Material

6.1 Description of Ketamine Assisted Psychotherapy

Ketamine Assisted Psychotherapy (KAP) in this paper describes a treatment utilizing ketamine at a subanesthetic dose, in a therapeutic office setting with a trained guide present for two hours, for intentional psycho-emotional healing purposes. KAP participants at the AIMS Institute proceed through medical and psychological screening for KAP eligibility, a prescription visit and a preparation visit prior to the ketamine experiential session where intention, or psychedelic *set* and *setting* are discussed. In this study, for the experiential sessions, ketamine was administered intramuscularly in the right or left deltoid, but can also be administered sublingually, orally or intravenously. Psychotherapeutic integration was provided 3-7 days following the ketamine experience, during which the facilitator/clinician and the participant discuss the content of the 'journey' experience and the resulting psycho- emotional or spiritual shifts.

6.2 Further Discussion

6.2.1 Decrease in NIH-HEALS Scores in One-Third

One-third (n=6) of participants had decreases to their psychosocial and spiritual metric score after their KAP sessions, indicating less positive responses to challenging life events. This includes participants #3, #5, #12, #15, #17, and #18 who had respective changes of -15, -6, -10, -5, -6, and -3 points to their NIH-HEALS scores. However, four of these six same participants (#3, #12, #17, #18) also reported a lower severity of depression symptoms with changes in PHQ-9 scores of -10, -4, -7,

and -6 points, respectively. Participants #3, #17, and #18 reported improvements in anxiety symptoms with reduction to GAD-7 scores by 18, 16, and 9 points, respectively. Participant #12 reported an increase in anxiety symptoms with GAD-7 scores increasing by 3 points, from 1 to 4. However, the participant's anxiety levels remained sub-clinical. Participants #5 and #15 did not complete baseline or follow-up GAD-7 or PHQ-9 scores. Participants #3, #5, and #15 experienced only one ketamine-assisted psychotherapy session in the given time period. Participants #12, #17 and #18 completed above-average number of ketamine sessions, at 3, 3, 4 respectively, and reported lower NIH-HEALS outcomes and improved PHQ-9 and GAD-7 scores.

<u>NIH-HEALS during the COVID-19 Pandemic: Community and Health Engagement</u>:
A possible explanation for this subset's trends are the impacts of the COVID-19 pandemic. During the 2020-2022 data collection period, the pandemic likely strained individuals' connections to supportive communities including spiritual and religious engagements. For some, the pandemic may also have changed their level of trust and confidence in their medical providers given the overburdened healthcare demand and emergence of new medical needs including care for long-COVID. These experiences of engagement and trust are queried in the NIH-HEALS questionnaire. Furthermore, several NIH-HEALS questions confront grief and mortality, feelings of helplessness, hopelessness, and fear around uncertainty. These emotions may have become more intense and pervasive during the pandemic.

Awareness of the pandemic's impacts of social isolation and loneliness drove a public health response with a primary focus of remedying barriers to communication (1). Through facilitating better interpersonal relationships, social technology use has been identified to reduce feelings of loneliness in older adults (2). Given the boom of technology to address pandemic-related distancing, increased access to lower-cost, low-barrier video-conferencing technologies may have allowed access for previously isolated members of the general population to improved social connection. Because there is no pre-pandemic NIH-HEALS data for this study population, some detected improvements in psychosocial and spiritual health may also be attributable to *greater* social connection during the pandemic. Together, these concepts imply, at the very least, a diversity of etiologies and contributing factors for depression and anxiety in a population with PSU and, therein, the need for individualized care plans.

<u>NIH-HEALS and Spiritual 'Remodeling':</u>
A decrease in NIH-HEALS score may also suggest that, for some people, KAP and the insights developed thereby can disrupt existing belief systems that are queried within NIH-HEALS. The consequences of a novel, mystical experience may in fact be neutral or beneficial to mood, but may run contrary to an individual's context of psychosocial and spiritual health as measured by the NIH-HEALS. The potential for spiritual 'remodeling' through mystical and entheogenic experience is generally considered a unique and attractive outcome within psychedelic-assisted psychotherapy. While some practitioners in the field may agree that this is inherently beneficial, the question arises regarding psychospiritual *risk* - risk of change or confrontation of prior beliefs. Individuals who elect

KAP should be informed that their existing models of psychospiritual understanding may shift after treatment, which may have unpredictable consequences detectable by NIH-HEALS.

Improvement in PHQ-9, GAD-7 Scores Despite Lower NIH-HEALS Score:
While six participants in our study experienced worsening NIH-HEALS scores by 3 to 15 points, four documented symptom improvements to anxiety, depression, or both, and we don't have data on the other two. This trend suggests that despite a decline in protective features of resilience during the pandemic for these participants, healing from depression and/or anxiety remained possible. It may also be that the NIH-HEALS questionnaire, compared to the GAD-7 or PHQ-9 questionnaires, more sensitively detects the direct impacts of the isolation and sociopolitical contexts of the pandemic or highlights areas where psychosocial and spiritual well-being is independent from mood.

6.3.1 Implications for Future Research - Continued

Comparing ACEs, Resilience Scores to KAP Response:
As a part of the AMOS and ACOS studies, Adverse Childhood Experiences scale and Resilience measures are collected as one-time assessments of risk and protective features. Future analysis is warranted to note any correlations between ACE or Resilience scores and response to KAP via psychometric changes. This type of study may provide insight into how a subset of individuals, based on ACE or Resilience scores, responds to KAP and how many KAP sessions are needed to sufficiently treat or manage mood concerns.

Improvements in GAD-7 and PHQ-9 scores prior to or in close proximity to KAP:
The linear trend lines used in analysis of psychometric response by number of days from ketamine experiential depict improvement in mood *prior to KAP* with greater proximity to the ketamine experience. Further studies with control groups receiving standard of care and/or psychotherapy alone may enhance our understanding of this phenomenon and thereby further inform the role of ketamine within existing models of care for PSU. Non-linear analyses of existing data may also be revealing as to individual patient change in scores based on proximity to KAP measured in days.

With a trend line composed of data both approaching and following the ketamine-assisted psychotherapy experiential sessions, future research may warrant evaluation of the mental health changes that occur in anticipating psychedelic therapy (Figures 2A, 2B, 2C). The AIMS Institute KAP Program includes several points of therapeutic contact prior to the ketamine session itself, including psychological and medical health screening visits, a prescription appointment, and a preparation visit. The improvement of psychometric scores leading up to the ketamine experience could reflect benefits from the therapeutic contact of these visits, or the role of expecting benefit from KAP (3, 4). The effect of these phenomena on mood and substance use outcomes warrant further investigation and analysis.

Supplementary Material References:

1. Trad NK, Wharam JF, Druss B. Addressing Loneliness in the Era of COVID-19. JAMA Health Forum. 2020;1(6):e200631. doi:10.1001/jamahealthforum.2020.0631

2. Chopik WJ. The benefits of social technology use among older adults are mediated by reduced loneliness. Cyberpsychol Behav Soc Netw. (2016) 19:551–6. doi: 10.1089/cyber.2016.0151

3. Kaertner LS, Steinborn MB, Kettner H, Spriggs MJ, Roseman L, Buchborn T, Balaet M, Timmermann C, Erritzoe D, Carhart-Harris RL. Positive expectations predict improved mental-health outcomes linked to psychedelic microdosing. Sci Rep. 2021 Jan 21;11(1):1941. doi: 10.1038/s41598-021-81446-7. PMID: 33479342; PMCID: PMC7820236.

4. Butler M, Jelen L, Rucker J. Expectancy in placebo-controlled trials of psychedelics: if so, so what? Psychopharmacology (Berl). 2022 Oct;239(10):3047-3055. doi: 10.1007/s00213-022-06221-6. Epub 2022 Sep 5. PMID: 36063208; PMCID: PMC9481484.
